# The role of TLR2 in exercise-induced immunomodulation in normal weight individuals

**DOI:** 10.1038/s41598-023-37811-9

**Published:** 2023-07-03

**Authors:** Fatemah Bahman, Halemah AlSaeed, Shaima Albeloushi, Fahd Al-Mulla, Rasheed Ahmad, Fatema Al-Rashed

**Affiliations:** 1 Immunology and Microbiology Department, Dasman Diabetes Institute, Al-Soor Street, P.O. Box 1180, 15462 Dasman, Kuwait; 2 Genetics and Bioinformatics Department, Dasman Diabetes Institute, 15462 Dasman, Kuwait

**Keywords:** Toll-like receptors, Interventional cardiology

## Abstract

Toll-like receptors (TLRs) have been targeted for therapeutic drug development for several disorders, including cardiovascular diseases (CVD), and diabetes mellitus. Daily levels physical activity (PA) has been purported to influence the systemic circulation of cytokines, affecting the overall activation of TLRs and influencing the inflammatory milieu. Objective and self-reported daily PA was tracked in 69 normal-weight adults. Freedson's cut-offs categorized daily PA intensity into the 25th lowest, medium, and top percentiles. Monocytic TLR2 expression was quantified by flow cytometry in fresh whole blood. Cross-sectional associations between flow cytometry measured TLR2^+^ subsets and clinical biomarkers were evaluated. PA increased circulation of TLR2^+^ monocytes. TLR2 expression was adversely corelated with reduced diastolic blood pressure (DBP), triglyceride (TG), and matrix metallopeptidase 9 (MMP9) levels. However, regression analysis indicated that only TG levels were independently linked with TLR2^+^ subsets in circulation in active participants. Higher daily levels of physical activity are associated with improved cardiovascular blood markers and elevated circulatory monocytic TLR2^+^ subsets. These findings suggest that TLR2 may play a role in modulating CVD risk factors in individuals leading physically active lifestyles.

## Introduction

Toll-like receptors (TLRs) are important immune system regulators, as they play a crucial role in the recognition of microbial pathogens^1^. TLRs can also recognize endogenous damage-specific molecular patterns produced by damaged cells^1,2^. This includes the recognition of host-induced molecules released in response to injury. Several studies have associated TLRs with myocardial survival, in particular the influence of both TLR4 and TLR2^3,4^.

Several groups have investigated the role of TLR2 in cardiovascular pathogenicity. However, different opposing outcomes have been reported, with some studies proposing that the activation of TLR2 can lead to the production of pro-inflammatory cytokines such as interleukin-6 (IL-6)^5^, tumor necrosis factor-alpha (TNF-α)^6^, interleukin-1 beta (IL-1β), vascular endothelial growth factor (VEGF)^7^, and matrix metalloproteinase (MMP9), which are known to be involved in the development of CVD^8–11^. Therefore, its depletion was found to be associated with favorable outcomes, especially in resolving the dysregulation of angiogenesis by inhibiting VEGF-MMP9 pathways^12^. While others proved that the depletion of soluble TLR2 released from circulatory monocytes exaggerated inflammatory responses that were found to associate with heart failure^13–15^.

Even though the causes of CVD are complicated, lifestyle factors like the amount of PA spent per day have a big effect on how the disease progress. In fact, regular PA has been shown to benefit the body by reducing inflammation and improving cardiovascular health^16^. Exercise is a well-known strategy for combating the chronic proinflammatory state triggered by metabolic syndrome^17^. Even though exercise was shown to modify TLR4 expression in some conditions in both humans and animals^18^, there is still no agreement on how exercise affects TLR2 regulation. Hence, the present study seeks to determine if variations in circulatory levels of TLR2, based on physical activity status, impact the evaluation of cardiovascular disease risk markers in healthy participants.

## Materials and methods

### Study design, participants, and anthropometric measurements

In this study, 69 individuals (27 men and 42 women) with an average age of 31.9 ± 4.5 years were recruited. All participants were healthy, lean adults with no past or current medical disorders and a BMI between 18.5 to 24.9 kg/m^2^. All participants were in good physical health with no disabilities preventing any physical movement. The study design included a total of two visits for each participant, with the first session informing participants about the study and the risks associated. Following clearance, participants were asked to sign a written consent form and were subjected to an extensive health screening. Each participant was given a wearable accelerometer to track physical activity for 7 days. Upon completion of the seven-day track, participants were asked for a second visit for anthropometric and biomedical assessments. The study utilized a uniform protocol for measuring the anthropometric parameters of all participants, and the equipment used for the assessments remained consistent throughout the research. A portable stadiometer was employed to measure the height of the subjects with an accuracy of 1.0 cm. Weight was measured to the nearest 0.2 kg with a standard physician’s beam scale (Detecto). Body mass index (BMI) was calculated as body weight in kilograms divided by height in meters squared (kg/m^2^). Participants were then taken to the phlebotomy unit for the collection of blood samples. Descriptive physical and biochemical statistics of the study participants stratified by gender are summarized in Supplementary Table 1. No significant difference was observed between male and female participants in terms of their age, BMI, biometrics, dietary intake (Supplementary Table 2), or their physical activity level (Supplementary Table 3). Since no significant differences were found between both gender subgroups regarding BMI and lipid profiles, subsequent analyses of male and female data were pooled. A written informed consent was obtained from all study participants and all methods are performed in accord with the ethical guidelines of the Declaration of Helsinki. The study was approved by Kuwait Ministry of Health Ethical Board (Approval ID#: 2017/542).

### Physical activity measurements

All participants were given an electronic accelerometer (Actigraph GT3X; Actigraph LLC, Pensacola, FL, USA). Subjects were advised to maintain their normal daily (habitual) physical activity levels during the study period. Accelerometers attached to elasticized belts were worn on right-side hips for seven consecutive days (except when bathing and during water activity). The actigraphy method enabled a reliable and objective assessment of daily physical activity^19,20^. The accelerometer provided physical activity measurements that included activity counts per min (CPM), vector magnitude, energy expenditure, step counts, activity intensity levels, and metabolic equivalents (METs). A 1-min epoch was used in this study, with activity counts assessed at 1-min intervals to ensure that the data quality for the participants includes at least four days during which the accelerometer was worn for at least 60% of the day. A non-wear time was taken as any block of time ≥ 60 min where the activity count was equal to zero. Freedson’s cut-offs^21,22^ were used to differentiate between the PA intensity levels including light-intensity physical activity (LPA) (100–1951 CPM), and moderate to vigorous-intensity activity (MVPA) (≥ 1952 CPM). All counts lower or equal to 99 CPM were considered as a sedentary status (SD). The data were also expressed as the mean intensity of each activity during the monitoring time i.e., total accelerometer counts per total monitoring time. For further analysis, participants were divided into three quartiles dependent on their physical activity level. The intensity of physical activity was divided into gross percentiles, where 25th (low PA identified as Q1), 50th (median PA identified as Q2) and 75th (high PA identified as Q3) percentiles were determined based on total count per min (CPM).

### Measurement of blood metabolic markers

Blood metabolic marker measurement was conducted as previously published^23^. Each participant's blood pressure and heart rate was measured before collecting blood sample for plasma isolation. The plasma was used to measure blood glucose, fasting insulin, cholesterol, high-density lipoprotein (HDL)-cholesterol, and triglyceride levels. Quality control sera were utilized to monitor the tests to guarantee accuracy and precision. The Homeostatic Model Assessment of Insulin Resistance (HOMA-IR) index was computed using the formula: HOMA-IR = fasting insulin (μU/L) × fasting glucose (nmol/L)/22.5.

### Flow cytometry for immune cell markers

**Figure 1:**
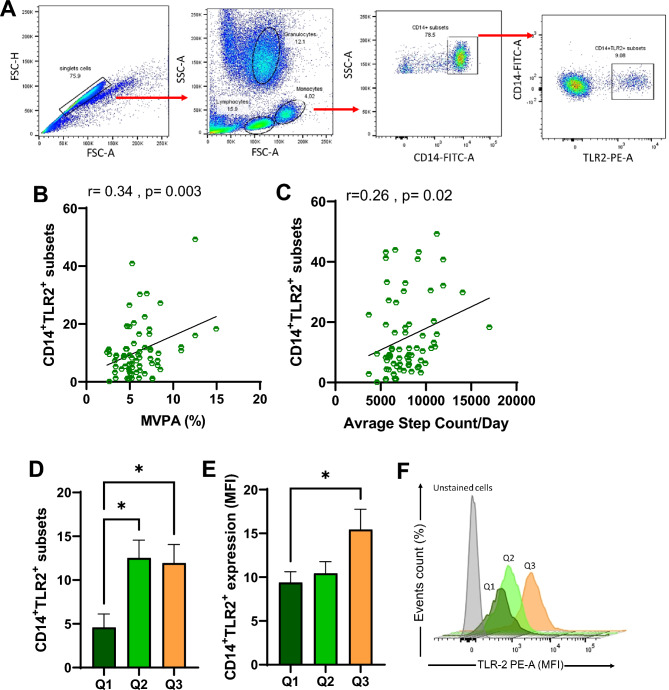
Physical activity and CD14^+^TLR2^+^ subset expression. Daily physical activity was tracked for each participant for an average of 7 days. The collected data was processed using Freedson’s cut-offs to differentiate between the PA intensity levels. Whole blood samples were lysed with lysing buffer and stained with anti-CD14-FITC and anti-TLR2-PE surface markers. (**A**) Shows gating strategy to identify CD14^+^TLR2^+^ subsets. Pearson’s correlation coefficient was conducted to investigate the relationship between (**B**) the level of CD14^+^TLR2^+^ subsets in circulation and the level of MVPA% spent per day. (**C**) CD14^+^TLR2^+^ subset expression and daily step count average. Totally daily physical activity was divided into three groups according to the 25% (Q1), 50% (Q2), and 75% (Q3) percentiles of CPM. Flow cytometry analysis was conducted to identify (**D**) total percentage count of CD14^+^TLR2^+^ subsets. Median florescence intensity (MFI) was calculated and presented in (**E**) bar graph and (**F**) representative histograms. All data are expressed as mean ± SD. p ≤ 0.05 was considered statistically significant (*p < 0.05, **p < 0.01).

To determine monocyte/macrophage lineages in the whole blood, flow cytometry analysis was conducted using freshly collected whole blood samples using the protocol previously published^24^. Briefly, 1 mL of lysing buffer was added to 0.1 mL of a blood sample to eliminate erythrocytes and the remaining peripheral blood mononuclear cells (PBMCs) populations were incubated with fluorochrome-conjugated mouse anti-human monoclonal antibodies against CD14, and TLR2 as well as with isotype-specific antibodies for respective controls. The gating strategy used in this study consisted of visualizing the cells on a forward scatter area FSC(A)/forward scatter height FSC (H) plot (Fig. 1A). A doublet exclusion gate was created to ensure singlet cells isolation. Gated singlet cells were further visualized on FSC(A)/side scatter SSC(A) plot to separate cells for their size and granulation to identify different leukocyte populations (lymphocytes, monocytes, and granulocytes). Based on forward and side scatter properties monocytes were identified. Selected gated monocytes cells were further displayed on SSC(A)/CD14^+^ to identify CD14^+^ subsets. TLR2^+^ subsets were then identified within CD14^+^ monocytes. The Median Fluorescence Intensity (MFI) of monocyte CD14^+^TLR2^+^ was calculated from the final gated population. Data were collected using a BD FACSCanto II flow cytometer. A minimum of ten thousand events were measured and saved for each sample. Data analysis was performed using FlowJo 10.8.1.

### Enzyme-linked immunosorbent assay (ELISA) for measuring soluble proteins and inflammatory cytokines/chemokines

Commercially available enzyme-linked immunosorbent assay (ELISA) kits were used for the detection of plasma insulin, C-peptide levels (Mercodia, Uppsala, Sweden) as well as circulatory levels of inflammatory cytokines/chemokines or factors including MMP9, and VEGF (R&D Systems, Minneapolis, MN, USA), following instructions from the manufacturers and the previously published protocol^25^.

### Statistical analysis

Data presented as mean ± SD. GraphPad Prism (version 6.05; San Diego, CA) and SPSS for Windows version 19.01 (IBM SPSS Inc., Armonk, NY) were used for statistical analysis. Multiple linear regression analysis was used to examine the relationship between monocyte surface marker expression and metabolic risk variables associated with longer periods of physical activity, adjusting for potential confounding factors. Pearson's correlation coefficient "r" was used to evaluate the linear association between variables. NS: not significant, *p < 0.05, **p < 0.01, ***p < 0.001, and ****p < 0.0001.

### Institutional review board statement

The study was approved by the Kuwait Ministry of Health (MOH) Ethics Board (2017/542).

## Results

### Higher moderate to vigorous-intensity activity levels is associated with higher circulatory CD14^+^TLR2^+^ subsets

The level and intensity of all participants’ physical activity were objectively evaluated using Freedson’s cut-offs^21^. On average, the participants spent 72.1 ± 4.8% of their day in a sedentary status (including sleeping time). While spending an average of 21.8 ± 4.0% of their day engaged in LPA movement and an average of 5.9 ± 2.3% of their day spent engaged in MVPA activity which is an equivalent of over 72 min per day. To understand if the level of daily physical activity had any modulatory effect on circulatory TLR2^+^ monocytes, participant peripheral blood cells were surface stained for TLR2 and analyzed by flow cytometry (Fig. 1A). On initial analysis, Pearson’s correlation investigation identified a positive significant association between the percentage of CD14^+^TLR2^+^ subsets in circulation and the level of MVPA% spent per day (Fig. 1B). Similar observation was found between the expression CD14^+^TLR2^+^ subsets and the average of step count taken per day (Fig. 1C). These observations suggest a possible interaction between the level of physical activity and the regulation of monocyte immune response through TLR2 receptor. To further investigate the intricacies of this interaction, the participants were divided into three groups depending on their physical activity level. General descriptive data across all three groups are presented in Supplementary Table 4 and Supplementary Table 5. Higher physical activity was found to be significantly associated with lower diastolic blood pressure (p < 0.04) and lower triglycerides (p < 0.04) with no other significant changes seen between groups. These results indicate an association between higher physical activity and CD14^+^TLR2^+^ subsets are associated with better cardiovascular health. Similar to our previous observation, both higher activity groups (Q2 and Q3) showed significant upregulation in monocytes’ surface expression of TLR2 receptor compared to the least active group (Q1) (Fig. 1D) with no significant difference between Q2 and Q3. However, when the median fluorescent intensity (MFI) was measured to evaluate the intensity of TLR2 receptor activation, it was found that only those with the highest 75% percentiles (Q3) were found to be significantly upregulated compared to Q1 (Fig. 1E,F).

### Individuals with higher MVPA and circulatory CD14^+^TLR2^+^ subsets show lower cardiovascular risk

**Figure 2:**
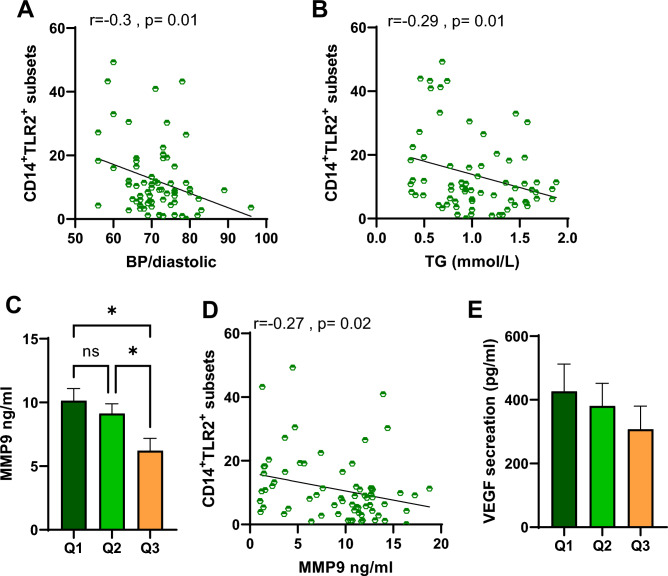
The association between CD14^+^TLR2^+^ subsets and both diastolic blood pressure and the cardiovascular risk factors. Pearson’s correlation coefficient was conducted to investigate the relationship between (**A**) diastolic blood pressure and circulatory CD14^+^TLR2^+^ subsets. (**B**) Plasma triglycerides and circulatory CD14^+^TLR2^+^ subsets. (**C**) MMP9 secretion in the serum. (**D**) Person correlation analysis for MMP9 secretion and CD14^+^TLR2^+^ subsets. (**E**) VEGF secretion in the serum. All data are expressed as mean ± SD. p ≤ 0.05 was considered statistically significant (*p < 0.05, **p < 0.01).

Interestingly, correlation analysis further suggested a negative significant association between circulatory CD14^+^TLR2^+^ subsets and both diastolic blood pressure and the level of triglycerides in the plasma (r = − 0.3, p = 0.01 and r = − 0.29, p = 0.01, respectively) (Fig. 2A,B). Both of those factors are known to be influenced by the monocytic secretion of cytokines, a process initiated through several pathways including the activation of the TLR2 receptor. In order to further examine this interaction, serum levels of metalloproteinase-9 (MMP9) and vascular endothelial growth factor (VEGF), two cytokines closely linked to cardiovascular health, were measured and compared. Higher physical activity groups Q2 and Q3 showed a significant reduction in MMP9 secretion in the serum compared to those in group Q1 (Fig. 2C). Person r association analysis further pointed out a significant negative relation between CD14^+^TLR2^+^ subsets and MMP9 secretion (r = − 0.27, *p*-value = 0.02) (Fig. 2D). Similar trend in reduction was also seen in VEGF secretion level. However, this reduction was not found to be significant (Fig. 2E).

Given that the level of diastolic blood pressure and the level of triglycerides and MMP9 in the plasma are all strong predictors of cardiac health, we next conducted multiple linear regression analyses to find if CD14^+^TLR2^+^ subsets are independently associated with them. Interestingly, our analysis showed that only triglycerides were found to be independently associated with CD14^+^TLR2^+^ subsets in circulation as seen in Table 1.

**Table 1:** Multiple linear regression analysis of the association between CD14^+^TLR2^+^ subsets with diastolic blood pressure, the level of triglycerides, and MMP9.

	CVD risk markers	Standardized coefficient β	95% Confidence interval	*p*-value
Monocyte CD14^+^TLR2^+^ (%)	BP/diastolic (mmHg)	0.2043	− 0.7417 to 0.07423	0.10
	Triglycerides (mmol/l)	3.400	− 13.94 to − 0.3620	0.03*
	Plasma MMP9 secretion (ng/ml)	0.3148	− 0.6652 to 0.5922	0.90

Statistical analysis: Pearson’s correlation coefficient, *p* < 0.05. (*) Significant differences between groups.

## Discussion

The present study examined the association between MVPA and the circulatory expression of TLR2 on monocytes. The influence of physical exercise on the modulation of immune function has prompted several research groups to investigate the effect of training on immune status^26–28^. Recent research has demonstrated that physical exercise may modify the immunological response, which is proposed to be mediated in part by TLR family members. Nevertheless, few studies have investigated the role of daily physical activity intensity in the regulation of monocyte and cytokine responses, particularly in the modulation of TLR2 expression.

In this study, we objectively tracked 7 days of habitual physical activity in 69 healthy, normal-weight adults with no past or current medical conditions. Followed by utilization of flow cytometry technology to identify the expression of TLR2^+^ circulatory monocytes. On a gross examination of the collected data, person r coefficient analysis indicated a positive association between the level and intensity of physical activity and the proportions of CD14^+^TLR2^+^ monocyte subsets in circulation. It’s not surprising that the increase in physical activity upregulates immune responses. This observation has been reported by several groups with indications of beneficial influence toward enhanced innate and adaptive immunity and better immune surveillance^29–32^. Indeed, a study presented by Kostka et al. showed a dose–response association between PA and infection risk, with a lower risk of infection reported in adults with higher levels of PA than those who were less active^29^; While emerging evidence from mouse models indicates that MVPA exercise prior to an infection improves host immunity by increasing the host's responsiveness to infectious pathogens^33^.

To further characterize the influence of PA on circulatory monocyte TLR2 expression, we divided our participants into three quartiles based on their CPM. According to the World Health Organization (WHO) guidelines of 2020, individuals are recommended to engage in a minimum of 75–150 min of MVPA per week to maintain general good health^34^. In our study cohort, we observed that the highly active groups (Q3 and Q2) engaged in an average of 107 min (7.4%) and 98 min (6.8%) of MVPA per week, respectively, while the least active group (Q1) had an average of 81 min per week (5.6%) (Supplementary Table 5). Interestingly, although both the high and low active groups fell within the WHO recommendation, our analysis revealed that individuals in the Q3 and Q2 groups, who demonstrated higher levels of physical activity (over 98 min of MVPA), exhibited favorable cardiovascular health trends. Specifically, they exhibited significantly lower diastolic blood pressure and lower triglyceride levels in their plasma compared to the Q1 group. These findings raise the possibility of reevaluating the WHO recommendations for PA levels, particularly concerning cardiovascular risk factors such as diastolic blood pressure and triglyceride levels.

Many compelling evidences support the therapeutic and protective effects of PA on the body, including the improvement of insulin sensitivity^35^, the alleviation of plasma dyslipidemia^36^, and the normalization of elevated blood pressure^37^. Interestingly, active individuals were also presented with higher CD14^+^TLR2^+^ cells in their circulation that were found to associate with both diastolic blood pressure and triglyceride plasma levels significantly and negatively. Previous work presented by a handful of researchers pointed out the influence of acute workout and the upregulation of TLR4 gene expression and the activation of the TLR4/NF-κB pathway^18,38,39^. However, the outcome of this activation remains debatable between induced damage and protective properties. The TLR4/NF-κB pathway is thought to be the major signal transduction mechanism involved in cardiomyocyte apoptosis, a common process in many cardiovascular events^40^. Though the activation of TLR/NF-κB, particularly under exercise settings, has been related to two opposite roles, with some research proposing cardiac damage triggered by prolonged expression of inflammation^41^ and others proposing cardiac-protective properties as it was shown that its upregulation is merely a participation in cardiac responses to physical exercise where its absence triggered myocardial injuries such as edema, hemorrhage, and inflammatory infiltration^39^. Even though all TLR signaling pathways culminate in activation of NF-κB, it remains unknown how TLR2/NF-κB is influenced when it comes to its cytokine’s expression in an exercise setting. Regardless, in the presented work, we found the upregulation of monocytic expression of TLR2 negatively associated with MMP9 and VEGF expression, even though the latter was found not to be significant. These data fall in line with the cardioprotective effect of the TLR signaling pathway. However, it’s important to point out that both the time and duration of TLR2/NF-κB expression might influence different outcomes similar to those reported under TLR4/NF-κB activation. It’s also important to point out that even though we have seen upregulation in circulatory monocytic expression of TLR2 under higher activity levels, we didn’t see any significant difference between Q2 and Q3, indicating controlled adaptation that is not dose dependent. We also did not record any significant change in inflammatory cytokine regulation beyond MMP9 (data are not shown). This could be explained in part by the healthy nature of our participants, but it’s also possible that the influence of TLR2 pathway activation is indirectly associated.

To test the dynamic of this relation, we conducted regression analysis to identify the variable with the most impact on a higher CD14^+^TLR2^+^ subset increase in circulation. The only independent explanatory variable for CD14^+^TLR2^+^ circulatory levels was the level of plasma triglyceride. Plasma triacylglycerols have long been associated with CVD and other cardiometabolic conditions^42^. In a study conducted by Castrillo et al., it was demonstrated that TLR activation suppresses liver X receptor (LXR) activity on its target genes^43^. LXRs are major transcription factors essential for cholesterol homeostasis and lipogenesis; it was demonstrated by several research groups that the upregulation of LXRα plays a critical function in triglyceride accumulation^44–46^. Regardless, whether monocytic TLR2 expression has any influence on LXRα activation remains controversial without proper mechanistic investigation, as it falls outside the scope of this presented work.

All in all, it is important to acknowledge certain limitations of our study. Although we had a sample of participants that were matched for their age, weight, and calorie intake, the sample size of 69 individuals remains relatively small. A larger sample size could provide more statistical power and enhance the generalizability of our findings. We also relied on a cross-sectional design, which limits the ability to establish causal relationships. Longitudinal studies with repeated measurements would be beneficial in understanding the temporal relationship between the intensity level of PA, circulatory TLR2 expression, and the associated cardiovascular parameters. We also relied on self-reported dietary logs. Even though collected data were carefully analyzed for dietary content and components, self-reported diaries are usually subject to recall bias or inaccuracies, which might mask the effect of diet on our measured parameters.

Regardless of these limitations, our study has yielded significant findings regarding the association between the intensity of PA, circulatory TLR2 expression, and cardiovascular blood markers. Not only does our finding solidify the protective role of physical activity in cardiovascular health in lean individuals, but it also provides a novel therapeutic target through the modulation of TLR2. Further research could explore the mechanistic pathways underlying this association, such as the impact of TLR2 activation on triglyceride levels and whether interventions targeting TLR2 signaling could have therapeutic implications for managing dyslipidemia. In conclusion, the presented work introduced the benefits of maintaining a physically active lifestyle. The findings from the presented data provide additional insight on how MVPA might influence monocytic expression of TLR2 and how this upregulation is associated with favorable outcomes, especially in cardiovascular health.

## Supplementary Information

Supplementary Tables.

## Data Availability

The datasets used and/or analyzed during the current study available from the corresponding author on reasonable request.
