# Supplementary material for: The role of TLR2 in exercise-induced immunomodulation in normal weight individuals
Source: Sci Rep. 2023 Jul 3;13:10703. doi: 10.1038/s41598-023-37811-9 (PMC10318094; doi:10.1038/s41598-023-37811-9)
Supplement: Supplementary file 1 — Supplementary Tables. [file 41598_2023_37811_MOESM1_ESM.pdf]

Supplementary Table 1 Descriptive characteristics of the study population stratified by sex

Physical characteristics of subjects	Male (n=27)	Female (n=42)	P-value
Age (years)	32.3 ± 4.4	31.6 ± 4.5	0.5456
Weight (kg)	68.1 ± 9.4	60.5 ± 9.9	0.0266*
Height (cm)	172.7 ± 9.9	161.3 ± 8.9	<0.0001****
BMI (kg/m2)	22.7 ± 1.7	22.5 ± 1.9	0.6135
Waist circumference (inch)	31.3 ± 3.0	30.1 ± 3.7	0.1738
Hip circumference (inch)	39.1 ± 4.1	40.1 ± 4.4	0.3219
BP/ systolic (mmHg)	112.6 ± 12.0	108.7 ± 9.7	0.1347
BP/diastolic (mmHg)	69.11 ± 8.8	70.3 ± 7.2	0.5437
HR	69.6 ± 11.2	71.8 ± 4.8	0.2700
Triglycerides (mmol/l)	0.8 ± 0.4	1.0 ± 0.4	0.1832
Total cholesterol (mmol/l)	4.7 ± 0.6	4.8 ± 0.5	0.4547
HDL cholesterol (mmol/l)	1.5 ± 0.3	1.4 ± 0.3	0.8295
Insulin Con. (mu/l)	4.2 ± 2.3	4.4 ± 1.9	0.7650
Fasting Glucose (mmol/L)	5.1 ± 0.6	4.8 ± 0.7	0.1045
HOMA-IR	0.9 ± 0.5	0.9 ± 0.4	0.8647
C-Peptide (pg/ml)	1.3 ± 0.4	1.2 ± 0.3	0.5893

All values are means ± standard deviations unless labeled otherwise

Supplementary Table 2 Descriptive dietary intake of the study population stratified by sex

All values are means ± standard deviations unless labeled otherwise

Dietary intake	Male (n=27)	Female (n=42)	P-value
**Total Calories (kcal)**	2039 ± 534.4	1916.2 ± 422	0.3146
**Carbs (g)**	255.8 ± 122.2	236.16 ± 60	0.4401
**Fat (g)**	57.1 ± 26.1	54.3 ± 22.4	0.7047
**Protein (g)**	75.1 ± 22.7	85.9 ± 50.3	0.3654
**Chol (mg)**	153.4 ± 119.8	236.0 ± 172.0	0.0716
**Sodium (mg)**	1189.5 ± 432.4	1094.7 ± 388.2	0.4749
**Sugars (g)**	61.1 ± 28.4	68.0 ± 32.6	0.3145
**Fibre (g)**	18.6 ± 15.6	19.0 ± 12.6	0.9320

Supplementary Table 3 Descriptive Physical Activity level of the study population stratified by sex

All values are means ± standard deviations unless labeled otherwise.

Physical Activity level	Male (n=27)	Female (n=42)	P-value
**Sedentary (%)**	72.7 ± 5.7	71.8 ± 11.0	0.4546
**Light intensity (%)**	21.1 ± 5.4	22.3 ± 3.5	0.2352
**Moderate intensity (%)**	5.1 ± 2.2	5.1 ± 1.9	0.9739
**Vigorous intensity (%)**	0.9 ± 0.6	0.7 ± 0.5	0.3486
**Moderate to Vigorous intensity (MVPA) (%)**	6.0 ± 2.6	5.9 ± 2.1	0.7967

Supplementary Table 4 Descriptive characteristics of the study population by quartiles

All values are means ± standard deviations unless labeled otherwise.

Physical characteristics of subjects by quartiles	Lowest 25% Q1	Mid-range Q2	Highest 75% Q3	P-value
	(n=18)	(n=27)	(n=42)	
**Age (years)**	32.7 ± 2.5	31.7 ± 2.9	33.48 ± 5.8	0.3587
**Weight (kg)**	67.0 ± 11.0	66.5 ±11.7	60.9 ± 8.7	0.0999
**Height (cm)**	168.8 ± 11.2	168.0 ± 11.1	163.4 ± 11.3	0.2249
**BMI (kg/m2)**	22.4 ± 1.9	22.4 ± 1.8	22.6 ± 1.6	0.9310
**Waist circumference (inch)**	31.0 ± 3.3	31.1 ± 3.0	31.2 ± 3.7	0.9772
**Hip circumference (inch)**	40.7 ± 4.6	40.2 ± 4.0	40.4 ± 4.6	0.9624
**BP/ systolic (mmHg)**	110.8 ± 8.9	110.1 ± 7.4	109.9 ± 13.2	0.9636
**BP/diastolic (mmHg)**	73.5 ± 7.5	69.1 ± 8.9	68.6 ± 5.9	0.0446
**HR**	73.7 ± 6.5	76.15 ± 6.6	73.7 ± 5.8	0.5479
**Triglycerides (mmol/l)**	1.1 ± 0.4	1.0 ± 0.4	0.8 ± 0.4	0.0483
**Total cholesterol (mmol/l)**	4.8 ± 0.6	4.8 ± 0.6	4.6 ± 0.4	0.3539
**HDL cholesterol (mmol/l)**	1.4 ± 0.4	1.5 ± 0.3	1.5 ± 0.4	0.7671
**Insulin Con. (mu/l)**	4.7 ± 2.0	4.2 ± 2.0	4.0 ± 2.1	0.5615
**Fasting Glucose (mmol/L)**	5.0 ± 0.4	5.0 ± 0.5	4.8 ± 0.9	0.4212
**HOMA-IR**	1.0 ± 0.4	0.9 ± 0.4	0.8 ± 0.4	0.4175
**C-Peptide (pg/ml)**	1.2 ± 0.2	1.2 ± 0.3	1.1 ± 0.3	0.6151

Supplementary Table 5 Descriptive Physical Activity level of the study population stratified by quartiles.

All values are means ± standard deviations unless labeled otherwise. Significance found between: Φ Q1 vs Q2, Δ Q1 vsQ3 , and ψ Q2 vs Q3.

Physical Activity level of subjects by quartiles	Lowest 25% Q1 (n=18)	Mid-range Q2 (n=27)	Highest 75% Q3 (n=42)	P-value
**Sedentary (%)**	72.5 ± 1.6	70.6 ± 1.1	67.2 ± 3.2	<0.0001Δψ
**Light intensity (%)**	21.8 ± 1.8	22.5 ± 1.5	25.1 ± 3.4	<0.0001Δψ
**Moderate intensity (%)**	5.0 ± 1	5.1 ± 0.9	6.3 ± 2.4	0.0213Δ
**Vigorous intensity (%)**	0.5 ± 0.4	0.6 ± 0.4	1.1 ± 0.7	0.0008Δψ
**Moderate to Vigorous intensity (MVPA) (%)**	5.6 ± 1.1	6.8 ± 0.9	7.4 ± 2.8	0.0024Δψ
